# *Elaeocarpus firdausii* (Elaeocarpaceae), a new species from tropical mountain forests of Sulawesi

**DOI:** 10.3897/phytokeys.62.7548

**Published:** 2016-03-25

**Authors:** Fabian Brambach, Mark Coode, Siria Biagioni, Heike Culmsee

**Affiliations:** 1 Ecology and Ecosystem Research, University of Göttingen, Untere Karspüle 2, 37073 Göttingen, Germany; 2 c/o Hannah Atkins, Royal Botanic Garden, 20A Inverleith Row, Edinburgh EH3 5LR, Scotland, UK; 3 Department of Palynology and Climate Dynamics, University of Göttingen, Untere Karspüle 2, 37073 Göttingen, Germany; 4 DBU Natural Heritage, German Federal Foundation for the Environment, An der Bornau 2, 49090 Osnabrück, Germany

**Keywords:** *Elaeocarpus*, Elaeocarpaceae, Indonesia, Lore Lindu National Park, tropical mountain forest, Sulawesi

## Abstract

Based on ongoing ecological research in mountain forests of Sulawesi, a new species, *Elaeocarpus firdausii* Brambach, Coode, Biagioni & Culmsee, **sp. nov.** is described and illustrated from mossy forests at > 2000 m and information provided on the species’ distribution, ecology and pollen morphology. *Elaeocarpus firdausii* is similar to *Elaeocarpus luteolignum* Coode but differs from the latter in having glabrous terminal buds, leaves with black gland dots, 4-merous, larger flowers, and more numerous stamens.

## Introduction

*Elaeocarpus* is the largest genus of the Elaeocarpaceae, comprising approximately 350 species in the Old World tropics and subtropics (excluding mainland Africa), from Madagascar and Mauritius in the west, to Japan in the north, Australia and New Zealand in the south and Polynesia including Hawai’i in the east (Coode 2004, Baba 2013). The greatest number of species is found in the Malesian region and taxonomic work on these is currently under way.

Some progress has been made in understanding infrageneric groupings: Already in the early 20^th^ century, Schlechter (1916) defined several sections for Papuasia, most of which still stand. Raymond Weibel worked on the whole genus, and made suggestions for sectional groupings, mostly in unpublished manuscripts at the Conservatoire in Geneva, copies of which have been put at the disposal of MC. In West Malesia (Sumatra, Peninsular Malaysia, Java, and Borneo), almost all species can be allotted to six major, morphologically defined groups. The “*Polystachyus* group” (Coode 1996c) is endemic to that area, while sect. *Acronodia* (Blume) Mast. (Coode 1996b) extends into the Lesser Sunda Islands. In Central and East Malesia, relationships are much less clear, although four of the groups from West Malesia are also represented here: sect. *Elaeocarpus* (Coode and Weibel 1994, Coode 1996a), sect. *Ganitrus* (Gaertn.) Brongn. & Gris (Coode 2010), sect. *Monocera* Mast. (Coode 2001c, 2007, 2014), and sect. *Coilopetalum* Schltr. (Coode 1978, 2001a).

Coode (1995) published 10 new species for Sulawesi. In this paper he drew attention to the contrast between Sulawesi and neighbouring Borneo: In Sulawesi, fewer species of *Elaeocarpus* are present (c. 70 in Borneo vs. c. 35 in Sulawesi), but they belong to a greater number of groups (6 in Borneo vs. ≥ 8 in Sulawesi). In addition to the four widespread groups mentioned above, three more with a more Eastern distribution are found: sect. *Dactylosphaera* Schltr. (Coode 1978), distributed from Sulawesi to New Guinea, sect. *Fissipetalum* Schltr. (Coode 1978, 2001b), from Sulawesi to Australia, and sect. *Oreocarpus* Schltr. (Coode 1978, 1984) which extends from the Philippines to Australia. Yet other species appear to be endemic, although their placement in any of the groups based on morphology has so far not been achieved. Coode (1995) suggested that some of these species from Sulawesi might be related to the *Polystachyus* group in Borneo.

Work on DNA samples at the Australian Tropical Herbarium (ATH), James Cook University in Cairns (e.g. Baba 2013), has established a molecular phylogenetic framework, within which, well-supported species-level relationships are beginning to emerge (Darren Crayn, ATH, personal communication).

**Figure 1.**
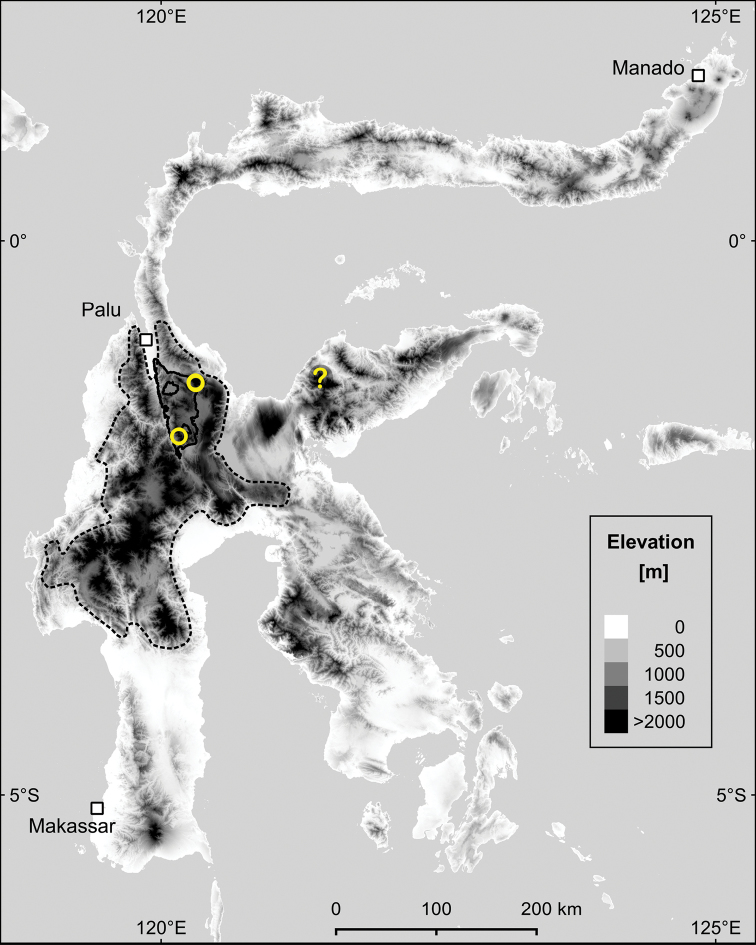
Map of known occurrences of *Elaeocarpus firdausii* in Sulawesi. Collecting localities are shown as yellow circles: Mt Rorekautimbu and Mt Malemo, both in Lore Lindu National Park (solid black line). The record on Mt Katopas on the Eastern peninsula (?) is based on a sighting without specimen. Most of the montane environments on the island are concentrated in the Central Sulawesi Mountains (CSM, dashed black line) stretching from near Palu into the Southern peninsula. Areas above 2000 m a.s.l. are shaded black. Map created with QGIS (QGIS Development Team 2015) using the digital elevation model of Jarvis et al. (2008).

Many of the recently described *Elaeocarpus* species from Sulawesi grow in montane forests above c. 1500 m (Coode 1995, 1996a, 2001a). Lore Lindu National Park (LLNP) is the protected area covering the largest portion of montane environments on the island. It is located within the large, contiguous upland area that occupies most of the central part of Sulawesi roughly between the city of Palu and the central part of the Southern peninsula (Fig. 1). We will refer to this area as Central Sulawesi Mountains (CSM) throughout the manuscript.

Recent research on tree diversity and composition in LLNP (Culmsee and Pitopang 2009, Culmsee et al. 2011), has improved our knowledge of the flora and vegetation of Sulawesi’s mountain forests. The continued research and new fieldwork in 2011–2012 have yielded material for 17 species of *Elaeocarpus*. One of them could not be assigned to any previously published species and is therefore proposed as new here.

## Methods

### Morphological observations

The relevant published identification keys for *Elaeocarpus* (Coode and Weibel 1994, Coode 1995, 1996a, 1996c, 2001a, 2001b, 2007) have been consulted, as well as herbarium collections of B, BO, GOET, K and L (herbarium acronyms follow Thiers (continuously updated): http://sweetgum.nybg.org/science/ih/) and online databases of digitized herbarium specimens (JSTOR 2015, RBG Kew 2015, Wieringa 2015). We also recorded the character states of our specimens in a DELTA matrix (Dallwitz et al. 2010) for *Elaeocarpus* in Malesia developed and maintained by MC. Most specimens of *Elaeocarpus* from the Malesian region in K and L have been seen by MC during his work on the genus over the last decades, so relatively few specimens remain unnamed. Our description is based solely on the material gathered during our (FB and HC) fieldwork in Central Sulawesi in 2011–2012, as no further matching specimens were found in herbaria. All our specimens were collected from permanently tagged trees in inventory plots. Duplicates of relevant specimens, including the type, were deposited in the Indonesian herbaria BO and CEB. All specimens seen by us for the description of the new species are marked with an exclamation mark in the present paper.

For the description, we boiled up flowers in dilute detergent for 5 minutes and dissected them afterwards. Dimensions were measured using a ruler with 0.5 mm accuracy. All colours and measures given refer to dried and pressed material unless stated otherwise. Photographs were taken in the field using a Canon EOS 500D camera with a Tamron AF 18-200mm f/6.2-38 lens.

Wood density (oven-dry mass per fresh volume) was determined from three wood cores extracted with increment borers and belonging to the specimens *Brambach et al. 0721*, *0973*, and *2041*, respectively. The samples’ fresh volume was measured by Archimedes’ principle and weight was noted from the same samples after oven-drying for 48h at 105°C.

### Pollen morphology

One closed flower bud (from the specimen *Brambach et al. 2041*) was processed for the description of the pollen morphology. The pollen grains were treated following standard Erdtman’s acetolysis method (Erdtman 1960, Faegri et al. 1989). The samples were mounted on permanent slides with Kaiser's glycerol gelatine and the slides were analysed under a Nikon Eclipse H550L photomicroscope at a magnification of 100×. Descriptions of the pollen grains were compiled following the terminology defined by Punt et al. (2007). The shape was described based on the measurements of the ratio between polar axis (P) and equatorial diameter (E) in equatorial view. Size values are based on a total of 10 grains measured, mean are shown with minima and maxima in parentheses.

### Conservation Assessment

With only three known collection localities (see “Distribution”), a meaningful calculation of the extent of occurrence (EOO) and area of occupancy (AOO) (IUCN Standards and Petitions Subcommittee 2014) as basis for the conservation assessment is not feasible. We, therefore, attempted to estimate the extent and location of potential habitats for the proposed species based on its known habitat preferences. For that, we used the CGIAR digital elevation model (Jarvis et al. 2008) in QGIS (QGIS Development Team 2015) to quantify land areas in Sulawesi above the elevation threshold of 2000 m. We defined this threshold based on our field observation of a marked and easily observable transition from mid-montane to upper montane (mossy) forest around this elevation. The proposed new species has so far only been recorded in upper montane forest at > 2000 m elevation (see “Habitat” below). We then assessed the forest condition at elevations ≥ 2000m using data from Cannon et al. (2007) and only used areas classified as “good” or “old-growth” by them for further analysis. As the proposed species has so far been recorded in the CSM and possibly the Eastern peninsula of Sulawesi, we also excluded all upper montane areas from the Northern and Southeastern peninsulas and the tip of the Southern peninsula (Fig. 1). The resulting potential habitats were used for the calculation of the extent of occurrence (EOO) and area of occupancy (AOO, grid cell size of 2 × 2 km) following the recommendations of IUCN Standards and Petitions Subcommittee (2014).

## Species description

### Elaeocarpus firdausii

Taxon classificationPlantaeOxalidalesElaeocarpaceae

Brambach, Coode, Biagioni & Culmsee sp. nov.

urn:lsid:ipni.org:names:77153914-1

**Figure 2.**
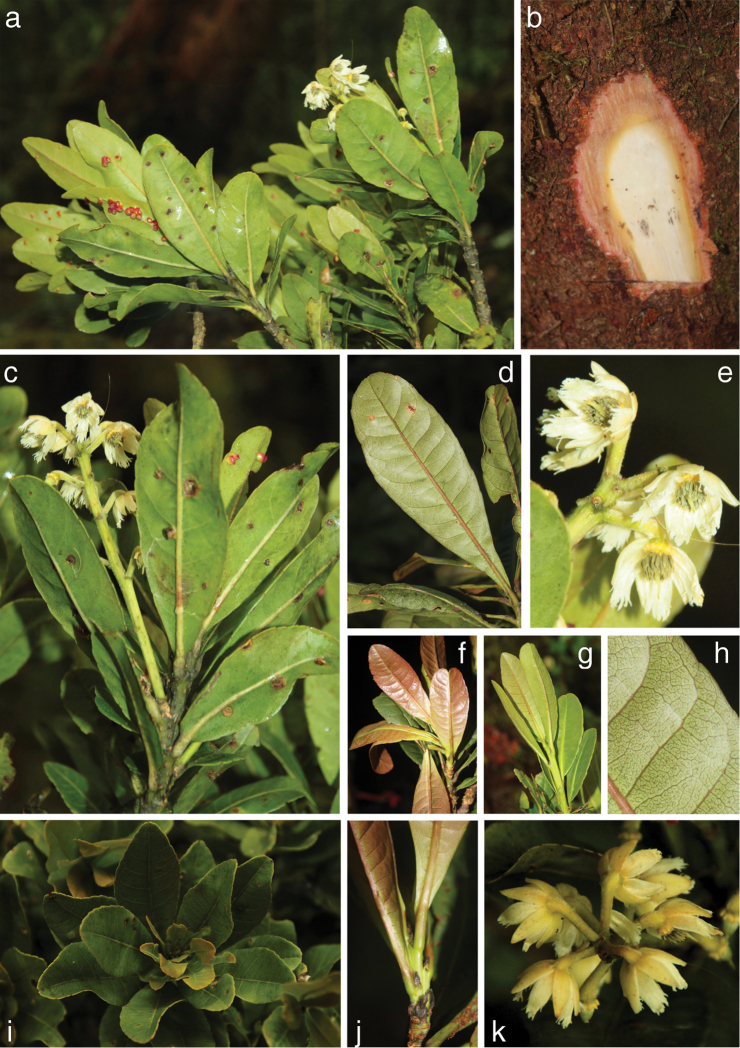
*Elaeocarpus firdausii*. **a** branch with leaves and flowers (note red leaf-galls) **b** bark slash **c** branch apex with inflorescence **d** underside of mature leaf **e** flowers with golden disc and grey anthers **f** reddish-brown young leaves **g** green young leaves **h** conspicuous reticulation on underside of mature leaf **i** clustered arrangement of leaves; **j** young twig with stipules **k** flowers on apically bent pedicels. **a**, **c**, **e**, **g**, **i**, and **k** from the type collection (*Brambach et al. 1953*); **b**, **d**, **f**, **h**, and **j** from *Brambach et al. 2041*.

**Figure 3.**
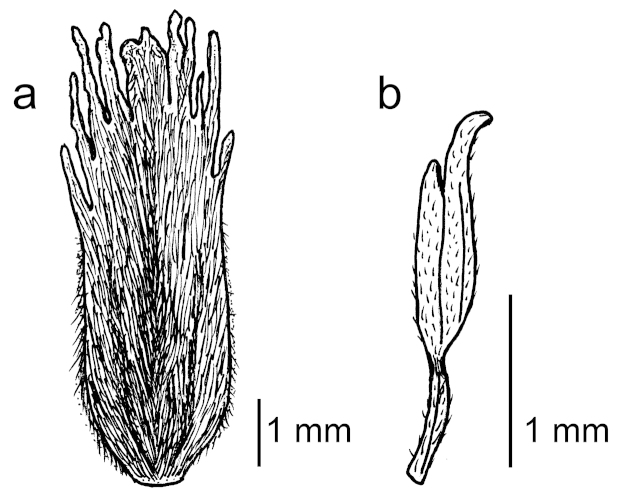
Flower details of *Elaeocarpus firdausii*. **a** petal with hairy outer surface and apical divisions **b** stamen with clearly longer outer anther-tooth to the right. Drawing by Heike Culmsee from *Brambach et al. 1953* (isotype, L!).

**Figure 4.**
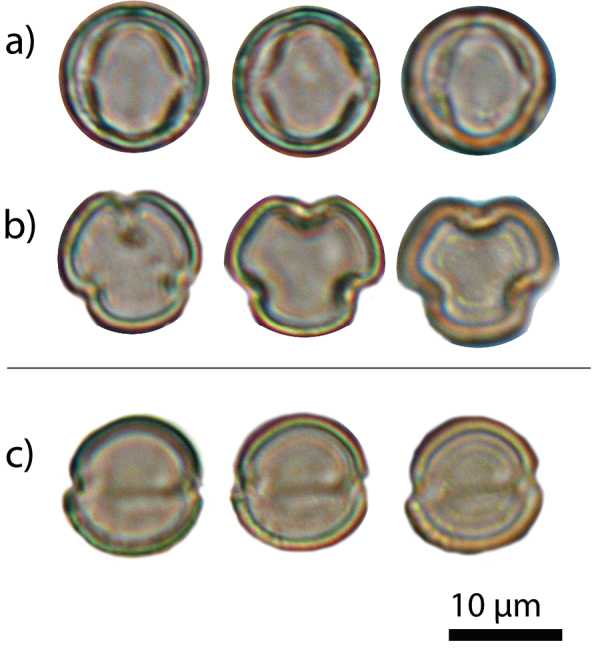
Pollen of *Elaeocarpus firdausii*. **a** the 3-colporate pollen type in equatorial view **b** same in polar view **c** the 2-colporate pollen type in polar view.

Figures 1 , 2 , 3 , 4

#### Diagnosis.

Similar to *Elaeocarpus luteolignum* Coode, but differing from that species in glabrous (vs minutely adpressed-hairy) terminal buds and young twigs, leaf blades with black gland dots (vs leaf blades without dots), 5-merous (vs 4-merous) flowers, larger flowers (e.g. sepals 5–8 × 1.5–2.5 vs 3–4 × 1.5 mm) and more numerous stamens (29–31 vs 20).

#### Type.

INDONESIA. Central Sulawesi (Sulawesi Tengah): Lore Lindu National Park, Kabupaten Poso, Kecamatan Lore Utara, 7.7 km NNE of village Sedoa, Mt Rorekautimbu, tree-inventory plot “Bulu Torenali”, 1°17.2'S, 120°18.7'E, 2350 m, 21–24 Apr 2012: *Brambach F, Mangopo H, Firdaus, Faber M, Tiranda R 1953* (flowers; holotype: K, 2 sheets, [K000720760]!, [K000720898]!; isotypes: BO (BO 1926842)!, CEB, L [L.2055441]!).

#### Description.

*Trees* 8–25 m tall, dbh ≤ 40 cm, without buttresses or stilt roots, flowering when full-grown. Outer bark reddish brown, verrucose; inner bark pinkish with white streaks, granular, innermost layer yellow, easily detachable from wood, wood cream to white.

*Twigs* glabrous, strongly angulate at first, later terete, twig bark longitudinally cracking, forming a net-like pattern, with large conspicuous leaf scars and many prominent lenticels, gummy-resinous where cut, 2.5–4.0 mm thick towards the tip, with gummy-resinous, glabrous terminal buds. *Stipules* caducous, linear-subulate to narrow-triangular, glabrous, often gummy, 1.5–5.0 mm long, tapering, entire.

*Leaves* spirally arranged, loosely to ± tightly grouped towards twig tips in older trees, in juveniles often scattered, appearing in flushes, leaves of one flush ± equal in size. Fresh leaves brownish-red when young, later dark green with contrasting paler midrib above, much lighter green and with contrasting darker green venation and the sometimes red midrib beneath, dying red. *Petioles* 2–14 mm long, 1–3 mm thick, glabrous or almost so, sometimes verrucose when mature, often longitudinally finely striate, usually flat in apical third above, sometimes rounded or slightly channelled above, distinct from or merging into decurrent leaf base (variable within a specimen), pulvinous or not on both ends, without pegs at apex, sometimes with elongate glands at the junction of petiole and lamina-margin, geniculate. *Blades* chartaceous to coriaceous, mostly oblong-obovate, some oblong-elliptic or obovate, 2.1–4.0 times as long as wide, (5–) 6–13 (–15.5) × 1.5–5.0 (–6.5) cm, acute to obtuse (80–110°) to rounded at apex, the very tip notched and with a (sometimes fused) pair of black glands, cuneate at base or tapering towards a broadly cuneate base (the larger leaves more narrowly cuneate), occasionally rounded, surface sometimes bullate, dull and glabrous above, glabrous or sometimes with some short adpressed hairs on the midrib beneath when young and then soon glabrescent, glabrous and not verrucose beneath when mature, with minute black gland dots on both sides. Midrib darker than lamina, prominent but widened and flattened towards base above, strongly prominent beneath, with 8–16 pairs of main lateral veins, diverging at 60–80° from midrib, straight for most of their length or curved, breaking up 3/4 to 7/8 inside margin, looping forward and mostly joining up; usually with intermediate veins in between, ± prominent and of same colour as or paler than lamina above and below, higher-order veins reticulate, obscure or ± clear and raised above and below, of same colour as lamina, areoles squarish, < 2 mm across, domatia absent. Margins ± entire to weakly glandular-serrate, sometimes less serrated in lower half, the teeth 2–11 mm apart, glands present regardless of serration, 0.5 mm long, spindle- or claw-shaped, sometimes elongate along margin, black.

*Inflorescences* in the axils of current leaves, solitary, racemose, ± of same length as subtending leaf, 3–8 cm long, axis angular, 1.2–1.5 mm thick at about halfway, with sparse, short, straight hairs between adpressed and spreading, 5–9-flowered.

*Flowers* bisexual, 5-merous (once 6-petalled), spiral or almost whorled on inflorescence, bracts early caducous, not seen, pedicels 6–18 mm long and 0.5–1 mm thick in flower, bent downwards and thickened at apex, buds ovoid, acute at apex. *Sepals* 5–8 × 1.5–2.5 mm, cream-coloured when fresh, not verrucose and ± pale adpressed-sericeous outside, densely white-velutinous next to the margins inside, otherwise short-sericeous inside but glabrous in the basalmost 1.5 mm, keeled inside for whole length. *Petals* thick and opaque, ivory-coloured on account of the hairs when fresh, oblong, parallel-sided almost to base, rounded to a narrow (1 mm wide) base, 6.5–7.5 mm long, 2.0–2.5 mm wide at widest point of limb, rounded at apex and divided into 9–12 narrow-triangular apical divisions 0.3–1.0 mm long, divisions unequal in length and grouped into lobes and acute at tip, not verrucose in dried material, densely white-sericeous outside, margins velvety or densely short-hairy throughout, densely short-hairy inside except for glabrous patch near base, with a low, narrow keel inside running for most of limb length, ± flat at midpoint and flat at base, without any infolding of margins. *Disk* golden when fresh, ± annular, 10-toothed, 0.5–0.8 mm high, densely covered with short, straight, golden hairs. *Stamens* 29–31, inserted in a ± single ring between disk and ovary; filaments 0.6–1.8 mm long, straight to somewhat incurved tapering from base to apex, glabrous or with a few minute hairs; anthers 1.6–2.5 mm long, khaki when fresh, minutely hairy, with outer tooth clearly much longer than inner and with a beak 0.2–0.5 mm long, beak glabrous or with a few minute hairs without setae at tip. *Ovary* placed above the disk, shape clearly narrowed at base, 2.0–2.5 mm long, densely short- to medium-hairy, 2–3-locular; ovules 8–12 per locule; style 2.5–3.5 mm long, stout, tapering to a point, glabrous except for the very base.

*Fruits* unknown.

#### Phenology.

Flowering was observed in April. No fruiting was observed.

#### Pollen morphology and dimorphism.

The pollen of *Elaeocarpus firdausii* is dimorphic as two distinct morphological pollen grains were observed in the sample. The most common one is a 3-aperturate pollen grain, typical of the family Elaeocarpaceae (Coode 2004). The second, less common (4%), type presents a 2-aperturate morphology and it is clearly distinguishable from the first (Fig. 4). The two pollen types are described as follows:

3-colporate type (Fig. 4a–b):

Prolate spheroidal to spheroidal pollen grains; outline in polar view (amb) rounded semi angular; psillate; P/E: 1.0 (0.9–1.1); polar axis (P): 12.2 (11.2-13.3) µm; equatorial axis (E): 11.9 (10.4-13.1) µm; apocolpium index 3–4 µm. Colpi 7.1–11.2 × 1–2 µm long with indistinct ends. Endoaperture lalongate, c. 1 µm in diameter. Exine c. 1 µm thick, sexine as thick as nexine.

2-colporate type (Fig. 4c):

Outline in polar view (amb) circular-elliptical; equatorial axis (E): 11.6 (10.5–12.7) µm. Remaining characteristics as the 3-colporate type.

So far, only one other case of pollen dimorphism has been documented for the genus *Elaeocarpus* (Huang 1972). In *Elaeocarpus firdausii*, the low percentage (ca. 4%) of the 2-colporate type as compared to the 3-colporate suggests the former is an aberrant morphology, possibly associated with hybridism as reported in other species (e.g. Bhowmik and Datta 2012).

#### Distribution.

Endemic to the central part of Sulawesi. The species is so far recorded with certainty from Mt Rorekautimbu and Mt Malemo at elevations from 2150 to 2400m (Fig. 1). Both mountains are located within LLNP. During our ecological fieldwork, we recorded *Elaeocarpus firdausii* in all three inventory plots at > 2000 m, although with rather few individuals at each site. Because of its apparent association with a distinct habitat (upper montane or mossy forest above c. 2000 m) and the general lack of information from montane habitats in Sulawesi, we consider it very likely that *Elaeocarpus firdausii* occurs in many of the upper montane forests of the CSM (Fig. 1).

FB observed a sterile sapling matching all vegetative characters of *Elaeocarpus firdausii* on Mt Katopas, c. 130 km east of the other two sites (1°12.7'S, 121°26.0'E, 2450 m, 6 Sep 2014), indicating a possible occurrence on Sulawesi’s Eastern peninsula (Fig. 1, question mark).

#### Habitat.

Based on the morphological information available *Elaeocarpus firdausii* is a regular component of upper montane (mossy) forests, where its individuals can form part of the canopy. These forests occur from c. 2000 m upwards in the LLNP area and are easily distinguished because of the dominance of conifers (Podocarpaceae, mostly *Dacrycarpus imbricatus* (Blume) de Laub. and *Phyllocladus hypophyllus* Hook.f.). They have a thick layer of epiphytic mosses and ferns on trunks and branches of the trees, ± abundant undergrowth, c. 20 m tall canopies with emergents reaching > 30 m and large amounts of dead wood. The soils are characterized by excess of moisture and heavy accumulation of organic matter. They were classified as Folic Gleysols, Folic Histosols and Folic Cambisols according to the WRB classification (IUSS Working Group WRB 2014). Dominant families besides conifers include Myrtaceae (e.g. *Syzygium* spp., *Xanthomyrtus angustifolia* A.J.Scott), Fagaceae (e.g. *Lithocarpus havilandii* (Stapf) Barnett), Paracryphiaceae (e.g. *Quintinia apoensis* (Elmer) Schltr.), and other Elaeocarpaceae (e.g. *Elaeocarpus steupii* Coode, Elaeocarpus teysmannii Koord. & Valeton subsp. domatiferus Coode).

#### Etymology.

The specific epithet honours our colleague Firdaus Dg. Matta (born 1984), formerly with Herbarium Celebense in Palu, Sulawesi, who collected the type specimen and contributed greatly to the success of our fieldwork with his skills in plant collection and identification.

#### Conservation status.

Based on the locations of the estimated potential habitat for *Elaeocarpus firdausii* we calculated an EOO of 58 534 km² and an AOO of 5 760 km². The latter is presumably an overestimate as not all potentially suitable sites will necessarily be occupied by the species. Nevertheless, occurrence over a relatively wide range is plausible, given the large distance (c. 55 km) between two of the collection sites. It is thus unlikely that either EOO or AOO will fall below the thresholds of criteria B1 or B2 for IUCN category VU (IUCN 2012). While deforestation is an ongoing threat to Sulawesi’s forests, upper montane forests are usually less affected because of their remote locations and difficult access (Cannon et al. 2007). Hence, we do not consider habitat destruction or exploitation by humans as an imminent threat to population levels. Given (1) the uncertainties in the estimated EOO and AOO, and (2) the recommendation to use a precautionary attitude in conservation assessments (IUCN Standards and Petitions Subcommittee 2014), we propose a preliminary extinction risk assessment of “Near Threatened” (NT) following the IUCN Red List Categories and Criteria (IUCN 2012).

#### Notes.

Based on the morphological information available, *Elaeocarpus firdausii* is probably related to *Elaeocarpus luteolignum*, *Elaeocarpus gambutanus* Coode and *Elaeocarpus linnaei* Coode; this assemblage may be sister to the *Polystachyus* group from Western Malesia.

In addition to the morphological differences between *Elaeocarpus firdausii* and *Elaeocarpus luteolignum* mentioned in the diagnosis above, according to our present knowledge there are differences in habitat preference: *Elaeocarpus firdausii* occurs in mossy forest at higher elevations while *Elaeocarpus luteolignum* is known from lower to mid-montane forest dominated by Fagaceae at 1200–1800 m (Coode 1995).

Both observations in the field and examination of dried specimens show that there are morphological differences between smaller understorey plants and mature canopy-forming individuals. The former have less-clustered, longer, thicker and relatively narrower leaves with more clearly bipulvinate petioles, less-rounded tips and more clearly serrate margins. We do not know whether these differences are related to age or rather to environmental factors, e.g. stronger radiation and transpiration in the canopy. Seedlings have even narrower leaves but the very short petioles are only swollen at the base. Conspicuous cup-shaped leaf galls or their presumed scars (Fig. 2) were present in all collected specimens. All sepals and petals have a glabrous patch at the base of the otherwise hairy inner surface. These glabrous portions are apparently pressed against the 10-lobed disc before anthesis. Wood density, based on three specimens, varied from 0.45–0.56 g/cm³.

#### Specimens examined.

Accession numbers are given in parentheses, barcode numbers in square brackets. Barcodes of specimens in K and L link to specimen records in the respective databases (RBG Kew 2015, Wieringa 2015).

INDONESIA. Central Sulawesi (Sulawesi Tengah), Lore Lindu National Park:

Kabupaten Poso, Kecamatan Lore Utara, 8.7 km NNE of village Sedoa, Mt Rorekautimbu, tree-inventory plot “Rorekautimbu”, 1°16.7'S, 120°18.6'E, 2400 m:

*Brambach F, Mangopo H, Firdaus, Faber M, Tiranda R 0721* (18–30 Jul 2011, sterile, BO (BO 1926844)!, CEB, GOET [GOET014481]!, K [K000720899]!, L [L.2055437]!);

*Culmsee H 2152* (Aug–Sep 2007, sterile, GOET [GOET014482]!).

Kab. Poso, Kec. Lore Utara, 7.7 km NNE of village Sedoa, Mt Rorekautimbu, tree-inventory plot “Bulu Torenali”, 1°17.2'S, 120°18.7'E, 2350 m, 21–24 Apr 2012:

*Brambach F, Mangopo H, Firdaus, Faber M, Tiranda R 2041* (flowers, BO (BO 1926843)!, CEB, GOET [GOET014478]!, K [K000720902]!, L [L.2055436]!);

*Mangopo H, Firdaus, Brambach F 11* (seedling, L [L.2055440]!).

Kab. Sigi, Kec. Kulawi Selatan, 7.7 km ENE of village Moa, Mt Malemo, tree-inventory plot “Tutu Malemo”, 1°45.9'S, 120°09.6'E, 2150 m, 18–23 Oct 2011:

*Brambach F, Mangopo H, Firdaus, Faber M, Tiranda R 0937* (sterile, CEB, GOET [GOET014480]!);

*Brambach F, Mangopo H, Firdaus, Faber M, Tiranda R 0998* (sterile, CEB, K [K000720900]!, L [L.2055438]!),

*Brambach F, Mangopo H, Firdaus, Faber M, Tiranda R 1026* (sterile, CEB, L [L.2055439]!),

*Brambach F, Mangopo H, Firdaus, Faber M, Tiranda R 1028* (sterile, CEB, GOET [GOET014479]!, K [K000720901]!).

## Supplementary Material

XML Treatment for Elaeocarpus firdausii

## References

[B1] Baba Y (2013) Evolution, systematics and taxonomy of *Elaeocarpus* (Elaeocarpaceae) in Australasia. Ph.D. thesis James Cook University http://researchonline.jcu.edu.au/38321/

[B2] Bhowmik S Datta BK (2012) Pollen Dimorphism of Several Members of Nymphaeaceae and Nelumbonaceae: An Index of Geographical and Ecological Variation. Notulae Scientia Biologicae 4: 38–44. doi: 10.15835/nsb.4.3.7689

[B3] Cannon CH Summers M Harting JR Kessler PJA (2007) Developing Conservation Priorities Based on Forest Type, Condition, and Threats in a Poorly Known Ecoregion: Sulawesi, Indonesia. Biotropica 39: 747–759. doi: 10.1111/j.1744-7429.2007.00323.x

[B4] Coode MJE (1978) A Conspectus of Elaeocarpaceae in Papuasia. Brunonia 1: 131–302. doi: 10.1071/BRU9780131

[B5] Coode MJE (1984) *Elaeocarpus* in Australia and New Zealand. Kew Bulletin 39: 509–20. doi: 10.2307/4108594

[B6] Coode MJE (1995) *Elaeocarpus* in the Flora Malesiana area: *E. kraengensis* and ten new species from Sulawesi. Kew Bulletin 50: 267–294. doi: 10.2307/4110631

[B7] Coode MJE (1996a) *Elaeocarpus* for Flora Malesiana: notes, new taxa and combinations in sect. *Elaeocarpus*: 2. Kew Bulletin 51: 83–101. doi: 10.2307/4118746

[B8] Coode MJE (1996b) *Elaeocarpus* for Flora Malesiana: notes, new taxa and combinations in the *Acronodia* group. Kew Bulletin 51: 267–300. doi: 10.2307/4119324

[B9] Coode MJE (1996c) *Elaeocarpus* for Flora Malesiana: the ‘*Polystachyus* group’. Kew Bulletin 51: 649–666. doi: 10.2307/4119720

[B10] Coode MJE (2001a) *Elaeocarpus* for Flora Malesiana: the *Coilopetalum* group in Sulawesi & Maluku. Kew Bulletin 56: 837–874. doi: 10.2307/4119298

[B11] Coode MJE (2001b) *Elaeocarpus* for Flora Malesiana: the *Fissipetalum* group in central Malesia. Kew Bulletin 56: 461–463. doi: 10.2307/4110966

[B12] Coode MJE (2001c) *Elaeocarpus* for Flora Malesiana: the *Verticellatae* subgroup of the *Monocera* group and a new Philippine species. Kew Bulletin 56: 885–901. doi: 10.2307/4119300

[B13] Coode MJE (2004) Elaeocarpaceae. In: Kubitzki K (Ed.) Flowering Plants. Dicotyledons: Celastrales, Oxalidales, Rosales, Cornales, Ericales. The Families and Genera of Vascular Plants. Springer, Berlin, Heidelberg, 135–144. http://link.springer.com/chapter/10.1007/978-3-662-07257-8_18

[B14] Coode MJE (2007) Elaeocarpaceae for Flora Malesiana: new information on *Elaeocarpus* from Borneo and Sulawesi. Kew Bulletin 62: 329–331. http://www.jstor.org/stable/20443358

[B15] Coode MJE (2010) *Elaeocarpus* for Flora Malesiana: new taxa and understanding in the *Ganitrus* group. Kew Bulletin 65: 355–399. doi: 10.1007/s12225-010-9223-2

[B16] Coode MJE (2014) *Elaeocarpus* for Flora Malesiana: the *Monocera* group in western Malesia. Kew Bulletin 69: 1–16. doi: 10.1007/s12225-014-9487-z

[B17] Coode MJE Weibel R (1994) *Elaeocarpus* for Flora Malesiana: notes, new taxa and combinations in sect. *Elaeocarpus*: 1. Kew Bulletin 49: 235–259. doi: 10.2307/4110262

[B18] Culmsee H Pitopang R (2009) Tree diversity in sub-montane and lower montane primary rain forests in Central Sulawesi. Blumea - Biodiversity, Evolution and Biogeography of Plants 54: 119–123. doi: 10.3767/000651909X47547

[B19] Culmsee H Pitopang R Mangopo H Sabir S (2011) Tree diversity and phytogeographical patterns of tropical high mountain rain forests in Central Sulawesi, Indonesia. Biodiversity and Conservation 20: 1103–1123. doi: 10.1007/s10531-011-0019-y

[B20] Dallwitz MJ Paine TA Zurcher EJ (2010) User’s guide to the DELTA system: a general system for processing taxonomic descriptions. 4th edition http://delta-intkey.com/www/uguide.htm

[B21] Erdtman G (1960) The acetolysis method. A revised description. Svensk Botanisk Tidskrift 54: 561–564.

[B22] Faegri K Kaland PE Krzywinski K (1989) Textbook of Pollen Analysis. 4th ed Wiley, Chichester (UK), 328 pp.

[B23] Huang T-C (1972) Pollen flora of Taiwan. National Taiwan University, Botany Dept. Press, Taipei, 297 pp.

[B24] IUCN (2012) IUCN Red List Categories and Criteria: Version 3.1. 2nd edition IUCN, Gland, Switzerland and Cambridge, UK, i-iv + 1–32 pp http://www.iucnredlist.org/documents/redlist_cats_crit_en.pdf

[B25] IUCN Standards and Petitions Subcommittee (2014) Guidelines for Using the IUCN Red List Categories and Criteria. Version 11. Prepared by the Standards and Petitions Subcommittee IUCN, 87 pp http://www.iucnredlist.org/documents/RedListGuidelines.pdf

[B26] IUSS Working Group WRB (2014) World Reference Base for Soil Resources 2014. International soil classification system for naming soils and creating legends for soil maps. FAO, Rome, 181 pp http://www.fao.org/3/a-i3794e.pdf

[B27] Jarvis A Reuter HI Nelson A Guevara E (2008) Hole-filled SRTM for the globe, Version 4. http://srtm.csi.cgiar.org [November 5, 2015]

[B28] JSTOR (2015) Global Plants on JSTOR. http://plants.jstor.org/ [December 15, 2015]

[B29] Punt W Hoen PP Blackmore S Nilsson S Le Thomas A (2007) Glossary of pollen and spore terminology. Review of Palaeobotany and Palynology 143: 1–81. doi: 10.1016/j.revpalbo.2006.06.008

[B30] QGIS Development Team (2015) QGIS Geographic Information System. Version 2.12.0. Open Source Geospatial Foundation Project. http://qgis.osgeo.org [December 15, 2015]

[B31] RBG Kew (2015) The Herbarium Catalogue, Royal Botanic Gardens, Kew Published on the Internet http://apps.kew.org/herbcat/gotoHomePage.do [December 15, 2015]

[B32] Schlechter R (1916) Die Elaeocarpaceen Papuasiens. Mit 9 Figuren im Text. Botanische Jahrbücher für Systematik, Pflanzengeschichte und Pflanzengeographie 54: 92–155. http://biodiversitylibrary.org/page/191244

[B33] Thiers B (continuously updated) Index Herbariorum: A global directory of public herbaria and associated staff. New York Botanical Garden’s Virtual Herbarium http://sweetgum.nybg.org/science/ih/ [March 17, 2015]

[B34] Wieringa J (2015) NHN Herbarium Database. National Herbarium of the Netherlands - NHN http://vstbol.leidenuniv.nl/nhn/Explore [December 15, 2015]

